# Balancing Innovation and Control: The European Union AI Act in an Era of Global Uncertainty

**DOI:** 10.2196/75527

**Published:** 2025-10-30

**Authors:** Elena Giovanna Bignami, Michele Russo, Federico Semeraro, Valentina Bellini

**Affiliations:** 1 Anesthesiology, Critical Care and Pain Medicine Division, Department of Medicine and Surgery, University of Parma, Via Gramsci, 14, Parma, 43121, Italy, 39 0521702111; 2 Department of Anaesthesia, Intensive Care and Prehospital Emergency, Ospedale Maggiore Carlo Alberto Pizzardi, Bologna, Italy

**Keywords:** EU AI Act, health care innovation, geopolitics, trade tariffs, regulatory sandboxes, AI ethics, supply chain resilience, European Union Artificial Intelligence, Artificial Intelligence ethics

## Abstract

The European Union's Artificial Intelligence Act (EU AI Act), adopted in 2024, establishes a landmark regulatory framework for artificial intelligence (AI) systems, with significant implications for health care. The Act classifies medical AI as "high-risk," imposing stringent requirements for transparency, data governance, and human oversight. While these measures aim to safeguard patient safety, they may also hinder innovation, particularly for smaller health care providers and startups. Concurrently, geopolitical instability—marked by rising military expenditures, trade tensions, and supply chain disruptions—threatens health care innovation and access. This paper examines the challenges and opportunities posed by the AI Act in health care within a volatile geopolitical landscape. It evaluates the intersection of Europe's regulatory approach with competing priorities, including technological sovereignty, ethical AI, and equitable health care, while addressing unintended consequences such as reduced innovation and supply chain vulnerabilities. The study employs a comprehensive review of the EU AI Act's provisions, geopolitical trends, and their implications for health care. It analyzes regulatory documents, stakeholder statements, and case studies to assess compliance burdens, innovation barriers, and geopolitical risks. The paper also synthesizes recommendations from multidisciplinary experts to propose actionable solutions. Key findings include: (1) the AI Act's high-risk classification for medical AI could improve patient safety but risks stifling innovation due to compliance costs (eg, €29,277 annually per AI unit) and certification burdens (€16,800-23,000 per unit); (2) geopolitical factors—such as United States-China semiconductor tariffs and EU rearmament—exacerbate supply chain vulnerabilities and divert funding from health care innovation; (3) the dominance of "superstar" firms in AI development may marginalize smaller players, further concentrating innovation in well-resourced organizations; and (4) regulatory sandboxes, AI literacy programs, and international collaboration emerge as viable strategies to balance innovation and compliance. The EU AI Act provides a critical framework for ethical AI in health care, but its success depends on mitigating regulatory burdens and geopolitical risks. Proactive measures—such as multidisciplinary task forces, resilient supply chains, and human-augmented AI systems—are essential to foster innovation while ensuring patient safety. Policymakers, clinicians, and technologists must collaborate to navigate these challenges in an era of global uncertainty.

## Introduction

We are writing to discuss the implications of the European Union’s Artificial Intelligence Act (EU AI Act) on health care, particularly in the context of the current geopolitical climate. The AI Act, which is poised to become a landmark regulation, has significant ramifications for the development, deployment, and governance of AI systems in health care. At the same time, the geopolitical landscape, marked by increasing military expenditures in Europe, the threat of trade tariffs from the United States, and broader global instability, demands that we consider how these regulatory frameworks interact with the realities of a rapidly changing world.

This paper aims to explore these complex dynamics, highlighting the opportunities and challenges posed by the AI Act in the context of a rapidly evolving geopolitical environment. By examining the intersection of AI regulation, health care innovation, and global instability, we seek to provide a nuanced understanding of how nations can navigate these challenges to ensure that AI technologies are harnessed responsibly and effectively for the benefit of patients and health care systems worldwide.

## The EU AI Act and Health care: A Brief Overview

The EU AI Act, adopted in 2024, represents a comprehensive attempt to regulate AI systems based on their risk levels [1]. For health care, the Act’s classification of AI systems as “high-risk” is particularly relevant, as it encompasses technologies that directly impact patient care and outcomes. High-risk AI systems in health care include those used in medical devices, patient management, and diagnostic tools. These systems are subject to stringent requirements, including robust risk management, data governance, and human oversight. The Act also mandates transparency, ensuring that health care providers and patients are aware when AI is being used in decision-making processes [2].

However, the Act’s focus on risk mitigation raises important questions about its potential impact on innovation. The health care sector is increasingly reliant on AI for tasks ranging from predictive analytics to robotic surgery [3,4], and the regulatory burden imposed by the AI Act could slow the pace of technological advancement. For instance, compliance with the Act requires significant investment in technical documentation, quality management systems, and cybersecurity measures [5]. While these requirements are essential for ensuring patient safety, they may disproportionately affect smaller health care providers or startups, particularly those developing or using high-risk AI applications such as diagnostic tools or patient management systems. Based on the “Study to Support an Impact Assessment of Regulatory Requirements for Artificial Intelligence in Europe” by the European Commission [6], compliance costs for a single AI unit could reach €29,277 (US $34,153) annually—a substantial burden for resource-constrained organizations. Startups may face additional challenges due to certification costs (€16,800‐23,000 per unit equivalent to US $19,598-26831 per unit) and the complexity of meeting requirements for human oversight, data governance, and transparency. While the regulation targets only 10% of AI systems as “high-risk,” health care innovations often fall into this category, potentially stifling innovation or diverting funds from research and development leading to a concentration of AI development in larger, well-funded organizations, potentially stifling competition and innovation However, the study notes that existing General Data Protection Regulation compliance (which overlaps with some AI Act requirements) might mitigate costs for some small and medium-sized enterprises, and sector-specific guidance could ease implementation. The lack of qualified notified bodies for certification may further delay market entry for smaller players.

Moreover, the current structure of the AI innovation ecosystem is marked by the dominance of “superstar” firms—large multinational technology corporations with extensive computational resources, proprietary data access, and deep regulatory expertise [7]. These firms are increasingly shaping the trajectory of AI development, including in the biomedical field, often through strategies of destructive creation that consolidate market power and marginalize smaller players. This dynamic has profound implications for health care AI, where dominant firms may prioritize scalable solutions that align with their commercial models rather than localized, need-based innovations. The AI Act’s high compliance costs, while necessary for risk mitigation, may unintentionally entrench this asymmetry by privileging those with existing infrastructure for regulatory conformity. This raises important questions about market concentration, regulatory capture, and the diminishing space for entrepreneurial and community-based innovation in digital health.

In addition, digital health tools and AI-based health care solutions possess attributes of nonrival public goods, generating substantial positive externalities and knowledge spillovers. For example, AI algorithms trained on diverse population datasets can yield insights far beyond their initial application, contributing to broader epidemiological surveillance, health equity research, and public health policy. However, the social benefits generated by these tools are not always aligned with the private incentives of developers. This misalignment risks underinvestment in socially valuable innovations—particularly those targeting rare diseases, marginalized populations, or preventive health—unless appropriate policy mechanisms are established. A nuanced regulatory framework must therefore, account not only for risks and compliance but also for the collective value of AI in health care and its role in a broader innovation commons.

Nevertheless, it is important to acknowledge that several regulatory bodies and major medical technology firms have responded positively to the AI Act, viewing it not as a barrier but as an enabler of innovation and trust [8]. Leading manufacturers, including multinational medical technology companies, have started developing internal AI governance frameworks aligned with the Act’s provisions, recognizing these as essential for building trust, enhancing market confidence, and maintaining global competitiveness. For instance, some companies have expressed support for classifying AI-based diagnostic tools as high-risk, arguing that clear regulatory requirements reduce legal uncertainty and promote safer, more consistent deployment at scale [9]. Regulatory authorities such as the European Medicines Agency and notified bodies have also underscored the value of early engagement through structured mechanisms like voluntary premarket consultations and regulatory sandboxes, which can facilitate compliance and shorten time-to-market for trustworthy AI-driven medical technologies [10].

Furthermore, adaptation strategies such as the integration of AI governance frameworks, including AI lifecycle management platforms and automated conformity assessment tools, are being actively adopted by industry leaders to meet the EU AI Act’s compliance requirements efficiently [11,12]. These tools facilitate adherence to obligations like risk management, data quality, technical documentation, human oversight, and post-market monitoring. Some hospital systems have also begun building dedicated AI regulatory and ethics teams, often working alongside AI developers and clinical stakeholders to ensure agile yet compliant innovation pipelines, particularly given the classification of many health care AI systems as ’high-risk’ under the Act [13]. These examples suggest that, while challenging, successful adaptation to the AI Act is feasible—especially for organizations that proactively invest in regulatory preparedness and multistakeholder collaboration.

## Geopolitical Uncertainty and Its Impact on Health Care

The geopolitical environment in which the AI Act is being implemented is fraught with uncertainty, and these external pressures could have profound implications for health care innovation. Europe is currently witnessing a significant increase in military expenditures, driven by the ongoing conflict in Ukraine and the broader rearmament of NATO member states. This shift in priorities has the potential to divert resources away from health care and other critical social services, including funding for AI research and development. In a context where health care systems are already under strain from rising costs and workforce shortages, this reallocation of resources could further hinder the adoption of AI technologies.

At the same time, the recent imposition of trade tariffs by the United States on key technology imports, including semiconductors and advanced medical devices, has exacerbated supply chain vulnerabilities. Many of the components essential for AI-driven health care technologies, such as semiconductors and advanced sensors, are produced in a limited number of countries, making supply chains vulnerable to geopolitical disruptions. For example, tensions between the United States and China over trade and technology could lead to export controls, making AI systems less accessible to health care providers in other countries.

Furthermore, the global competition for AI dominance, often framed as a race between the United States and China, has significant implications for Europe’s ability to maintain its technological sovereignty [14]. While the AI Act represents a step forward in terms of ethical AI governance, it may also be seen as a barrier to innovation if it is not balanced with sufficient support for research and development. Europe risks falling behind in the global AI race if its regulatory frameworks are perceived as overly restrictive, potentially leading to a “brain drain” of AI talent to more innovation-friendly regions.

## AI, Labor Markets, and Indirect Health Risks

The transformative potential of generative and autonomous AI systems extends beyond clinical applications and into the broader socioeconomic fabric, including labor markets, which are inextricably linked to public health. The automation of diagnostic processes, administrative workflows, and even clinical reasoning threatens to displace certain categories of health care employment. While AI can augment clinical tasks and reduce burden, its large-scale deployment without social protections may contribute to job insecurity, professional deskilling, and psychological stress among health care workers [15]. Drawing from the work of Case and Deaton on “deaths of despair” [16], we recognize that economic precarity and disconnection from meaningful work are major determinants of population health. Policymakers must anticipate these secondary effects when promoting AI adoption and consider protective strategies such as retraining programs, mental health support, and frameworks for meaningful human-machine collaboration in clinical settings.

## The Intersection of AI Regulation and Geopolitical Realities

The intersection of AI regulation and geopolitical realities presents both challenges and opportunities for health care. On the one hand, the AI Act provides a framework for ensuring that AI systems in health care are safe, transparent, and accountable. This is particularly important in a field where decisions can have life-or-death consequences. On the other hand, the Act must be implemented in a way that does not hinder the ability of European health care providers to compete globally or to respond to emerging threats, whether they be pandemics, cyberattacks, or the consequences of geopolitical instability.

One area where the AI Act could have a particularly significant impact is in the development of AI-driven diagnostic tools. These tools, which have the potential to revolutionize health care by enabling earlier and more accurate diagnoses, are classified as high-risk under the Act [17]. While this classification is understandable given the potential consequences of diagnostic errors, it also raises questions about how to balance regulation with innovation. For example, how can we ensure that the regulatory burden does not discourage the development of AI tools that could improve access to health care in underserved areas? This is particularly relevant in the context of global health disparities, where AI-driven diagnostics could play a crucial role in bridging gaps in health care access.

Additionally, the geopolitical context adds another layer of complexity to the implementation of the AI Act. For instance, the increasing use of AI in military applications, such as autonomous drones and cyber warfare, raises ethical questions about the dual-use potential of AI technologies. While the AI Act focuses on civilian applications, the broader geopolitical environment may influence how these technologies are developed and deployed. Europe must navigate these challenges carefully to ensure that its regulatory frameworks do not inadvertently weaken its position in the global AI landscape.

Another dimension often overlooked in discussions of AI governance is the environmental impact of AI development and its intersection with health. High-performance computing, data storage, and large-scale model training required for cutting-edge health AI systems consume significant energy and contribute to carbon emissions. These environmental costs carry indirect health consequences, particularly for vulnerable populations already burdened by climate-related disease and resource scarcity. In this light, responsible AI innovation in health care must also be environmentally sustainable [18]. Future iterations of the AI Act and related frameworks could benefit from incorporating sustainability criteria—such as lifecycle assessments, emissions reporting, or green AI standards—to ensure that health innovation does not inadvertently generate new public health risks through environmental degradation.

## A Call for Reflection and Action

In light of these challenges, we urge the scientific health care community to take a proactive role in addressing the intersection of AI regulation, health care, and geopolitical uncertainty. The rapid evolution of AI technologies, coupled with increasing global complexity, demands a coordinated and realistic response. While some solutions may face implementation challenges, they can serve as guiding principles or pilot initiatives adaptable to local conditions. To navigate this landscape effectively, we propose the following revised and context-aware actions.

### Develop Context-Aware Multidisciplinary Task Forces

While political and institutional fragmentation across the EU poses challenges, the creation of multidisciplinary task forces—initially on a national or regional level—can serve as a pragmatic starting point. These groups, comprising AI experts, clinicians, ethicists, and legal scholars, can work to identify actionable bottlenecks in the implementation of the AI Act. Rather than replacing the EU’s legislative role, such task forces would serve in an advisory capacity, promoting dialogue between practitioners and regulators. Initiatives like the European AI Alliance show that inclusive, multistakeholder discussion is possible despite complex politics [19]. One promising approach is the creation of regulatory sandboxes—controlled testing environments where AI technologies can be piloted under temporary regulatory flexibility, with close oversight from authorities. For instance, a hospital could trial an AI diagnostic tool under monitored conditions, with safeguards like mandatory human oversight, real-time bias audits, and strict patient consent protocols. Such sandboxes, already tested in sectors like finance (eg, the UK’s Financial Conduct Authority’s sandbox for fintech) and digital health (eg, the Food and Drug Administration’s precertification program), could accelerate compliance while mitigating risks [20,21]. By adapting these models to health care, task forces could help shape sandbox frameworks that align with the AI Act’s high-risk requirements—ensuring innovation thrives without compromising safety or ethical standards.

### Invest in AI Literacy and Training

To equip health care professionals with the knowledge and skills needed to understand and work alongside AI systems, investment in AI literacy could be beneficial. This includes training on the ethical use of AI, data governance, and cybersecurity, ensuring that health care providers can confidently integrate AI into their practices while adhering to regulatory requirements [22]. Training programs should also address the geopolitical implications of AI, helping health care professionals understand how global trends may impact their work. Given the linguistic and cultural diversity across Europe, a fully centralized training system is indeed unrealistic. Instead, we advocate for a federated approach by developing modular training curricula that can be localized and adapted by individual Member States or health care institutions. EU agencies such as the European Health and Digital Executive Agency [23] can support these initiatives by setting minimum competency standards and offering open-access multilingual materials. This approach balances regional autonomy with EU-wide quality assurance and can be complemented by peer-exchange platforms and international collaboration.

### Advocate for Geopolitically Resilient Supply Chains

It includes working with policymakers and industry leaders to create resilient supply chains for medical devices and AI technologies. This includes diversifying suppliers, investing in local manufacturing capabilities, and developing contingency plans to mitigate the impact of trade tariffs or geopolitical disruptions. However, we recognize that building a completely geopolitically resilient supply chain is not immediately feasible. Instead, we propose diversification and strategic redundancy as realistic steps. This includes identifying critical dependencies (eg, on semiconductors), incentivizing dual-sourcing contracts, and fostering EU-based innovation hubs, such as the European Chips Act initiative [24]. While resilience cannot eliminate risk, it can reduce vulnerability to single points of failure, particularly in high-risk sectors like health care.

### Foster International Collaboration Without Sacrificing Sovereignty

Despite growing techno-nationalism, there remains scope for international collaboration on standards and ethics—especially in health, where global challenges demand collective solutions. Forums such as the World Health Organization, Organisation for Economic Co-operation and Development, and G7/G20 AI initiatives offer platforms for aligning regulatory principles without ceding national control [25-27]. Shared frameworks can lower regulatory friction and support equitable access to AI-driven health care in lower-resource settings.

### Implement Human-Augmented AI Systems

Adopting a “human-in-the-loop” approach where AI-generated responses are verified by vetted peer clinician consultants might be . This approach combines the efficiency of AI with the trust and expertise of human clinicians, ensuring more accurate and reliable decision support. The system can be scaled from local institutions to global platforms, with various incentives for peer consultants, such as recognition, continuing medical education credits, or monetary compensation [28]. Of course, it is not always practical or necessary to require human oversight for every AI output. However, for high-stakes clinical applications (eg, diagnosis, triage, treatment decisions), human-in-the-loop systems offer a safeguard against automation bias and unexpected model behavior [29]. We suggest developing risk-tiered guidelines, where human oversight is proportionate to potential patient harm. Enforcement can occur through compliance mechanisms tied to medical device regulation and certification (eg, CE marking), mirroring how pharmacovigilance currently mandates oversight for novel therapies [30].

### Prioritize Ethical AI Development

Establishment of clear ethical guidelines for the use of AI in health care, particularly in high-stakes scenarios such as triage during conflicts or pandemics can further development. These guidelines should address issues such as bias, transparency, and accountability, ensuring that AI systems are used in ways that prioritize patient welfare and uphold fundamental rights. Ethical considerations should also extend to the geopolitical implications of AI, such as the potential for AI technologies to be used in ways that exacerbate global inequalities. Regulating ethical AI development in private industry is undeniably challenging, especially without global harmonization. However, governments and institutions can shape behavior through a mix of regulation, incentives, and transparency requirements. This includes public reporting of algorithmic impact assessments, bias audits, and governance structures—similar to financial disclosures. Procurement policies can also serve as a powerful lever: health care systems can favor vendors that demonstrate ethical compliance, much like environmental or labor standards in other sectors [31]. Furthermore, ethical guidelines co-developed with industry stakeholders can foster shared ownership and practical relevance.

**Figure 1.**
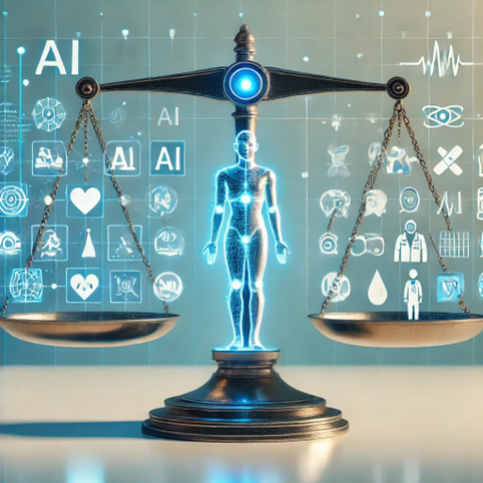
AI-generated image representing the need for a balanced approach to AI regulation that safeguards patient safety while fostering innovation, even in the face of a rapidly changing geopolitical landscape. AI: artificial intelligence.

By framing these actions not as universal solutions but as scalable, adaptable strategies, we believe the scientific health care community can meaningfully engage with both the risks and promises of AI. Realistic implementation pathways grounded in the current political, linguistic, and economic landscape are essential for ensuring that AI technologies contribute to safe, equitable, and resilient health care systems across Europe and beyond. (Figure 1)

## Conclusion

The EU AI Act offers a vital framework for ensuring the safe and ethical use of AI in health care but its effectiveness depends on balanced implementation amid geopolitical uncertainty. To avoid stifling innovation, regulatory efforts must be paired with support for adaptation, investment in AI literacy, and resilient infrastructure. To maximize the benefits of the EU AI Act while mitigating its risks, a broadened perspective is needed—one that recognizes not only direct clinical applications but also the wider socio-economic and environmental contexts in which health care AI operates. This includes addressing market concentration in AI research and development, aligning private innovation with public health needs, anticipating labor market disruptions, and reducing the ecological footprint of digital health. By integrating these factors into future policy and implementation strategies, Europe can foster an AI ecosystem that is not only safe and innovative, but also equitable, sustainable, and socially responsible.

We urge policymakers, health care leaders, and industry to collaborate on practical solutions, such as regulatory sandboxes, ethical oversight, and international alignment to ensure that AI advances benefit patients while reinforcing Europe’s role in global health innovation.

## Abbreviations

AI: artificial intelligence
EU AI: European Union's Artificial Intelligence
